# An atypical endoscopic treatment for “Buried Bumper Syndrome”

**DOI:** 10.1055/a-2381-4938

**Published:** 2024-09-04

**Authors:** Matei-Alexandru Cozma, Maxime Saunier, Arthur Berger, Frank Zerbib

**Affiliations:** 1 Gastroenterology and Hepatology Department, Colentina Clinical Hospital, Bucharest, Romania; 2 Gastroenterology, Hepatology and Digestive Oncology Department, Haut-Lévêque Hospital, Bordeaux, France

Percutaneous endoscopic gastrostomy (secured using a balloon or by a bumper system) is a widely used method for feeding and nutritional support in patients requiring long-term enteral nutrition. Although considered a safe method, complication rates vary from 0.4% to 22.5% of cases 1 . Buried bumper syndrome (BBS) represents a rare but important complication, with an incidence between 0.3% and 2.4%, and is defined as the migration of the internal bumper anywhere between the gastric wall and the skin, along the gastrostomy tract 2 . Management can be difficult. Historically represented by surgical methods, management now involves a variety of endoscopic devices, including needle-knives, wire-guided papillotome, or the Flamingo-type sphincterotome 3 .

We present the case of a 62-year-old patient who underwent gastrostomy with a bumper fixation system by the peroral pull technique (the Ponsky method), during prolonged hospitalization for cardiorespiratory arrest secondary to acute myocardial infarction. Owing to low compliance and repeated attempts of self-extraction, the gastrostomy was removed using the “cut and push” method. Afterwards, the patient presented with cutaneous discharge of gastric fluid, painful induration, and superficial periorificial ulceration. Diagnosis of BBS was suspected following a computed tomography, which highlighted the persistence of a foreign body in contact with the anterior gastric wall ( Fig. 1 ), and confirmed by upper endoscopy, which showed the presence of a 2-cm submucosal lesion, with a central millimetric orifice and continuous purulent discharge ( Fig. 2 ).

**Fig. 1:**
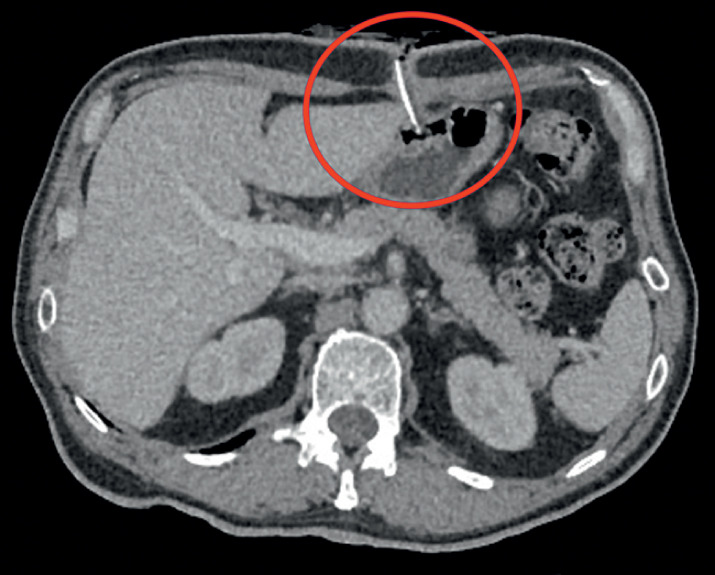
Computed tomography image of the internal bumper of the gastrostomy in the anterior abdominal wall.

**Fig. 2:**
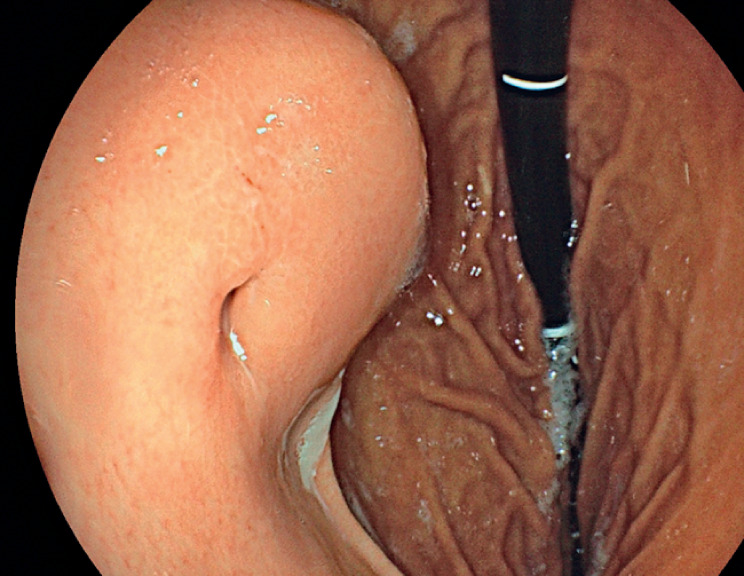
Endoscopic view of the submucosal cavity containing the internal bumper of the gastrostomy.

The removal technique consisted of endoscopic submucosal dissection (ESD) using a 2.0 DualKnife device (Olympus, Tokyo, Japan), assisted by an elastic traction system. Dissection further exposed the buried bumper, which was later removed using a simple biopsy forceps. Finally, the remaining cavity was closed with four hemostatic clips ( Video 1 ). We report no intra- or post-procedural complications during the 3-month endoscopic follow-up.

Dissection of the abscessed cavity in the anterior gastric wall, followed by successful removal of the internal bumper and closure of the incision.Video 1

Multiple cases describing ESD as a treatment method for BBS are reported in the literature 4 5 . Advantages include a low complication rate and a short recovery time. In experienced centers, ESD appears to be a safe, effective, and less invasive option for the treatment of BBS.

Endoscopy_UCTN_Code_CPL_1AH_2AI

## Notes

Endoscopy E-Videos https://eref.thieme.de/e-videos
*E-Videos* is an open access online section of the journal *Endoscopy* , reporting on interesting cases and new techniques in gastroenterological endoscopy. All papers include a high-quality video and are published with a Creative Commons CC-BY license. Endoscopy E-Videos qualify for HINARI discounts and waivers and eligibility is automatically checked during the submission process. We grant 100% waivers to articles whose corresponding authors are based in Group A countries and 50% waivers to those who are based in Group B countries as classified by Research4Life (see: https://www.research4life.org/access/eligibility/ ). This section has its own submission website at https://mc.manuscriptcentral.com/e-videos .
